# Polyvinylidene Fluoride-Based Metasurface for High-Quality Active Switching and Spectrum Shaping in the Terahertz G-Band

**DOI:** 10.3390/polym13111860

**Published:** 2021-06-03

**Authors:** Octavian Danila

**Affiliations:** Physics Department, ‘Politehnica’ University of Bucharest, 060042 Bucharest, Romania; octavian.danila@upb.ro

**Keywords:** polyvinylidene fluoride, frequency-selective surface, terahertz sensing, terahertz imaging

## Abstract

We report theoretical investigations performed in the terahertz G-band, in the 228–232 GHz spectral window for a piezoelectrically-responsive ring-cone element metasurface composed of polyvinylidene fluoride (PVDF)/Silicon and PVDF/Silica glass. The choosing of this spectral window is motivated by a multitude of applications in terahertz detection and terahertz imaging, that commonly make use of this band. The uniqueness of the envisioned architecture resides in the combination between the readily-available polyvinylidene fluoride polymer and silicon/silica glass substrates, together with the introduction of an extra degree of freedom, in the form of a ring-cone architecture, and the active control of the geometric sizes through the longitudinal piezoelectric effect exhibited by the polymer. The spectral response of the metasurface is dependent on the combination between the polymer elements and the substrate, and ranges from near-zero absorption switching to a resonant behavior and significant absorption. The interaction between the electromagnetic field and the polymer-based metasurface also modifies the phase of the reflected and transmitted waves over a full 2π range, permitting complete control of the electric field polarization. Moreover, we take advantage of the longitudinal piezoelectric effect of PVDF and analyze the spectrum shaping capability of the polymer-based metasurface. Our analysis highlights the capability of the proposed architecture to achieve complete electric field polarization control, near-zero optical switching and resonant behavior, depending on the geometries and sizes of the architecture elements resulting from construction considerations and from the externally applied voltages through the piezoelectric effect.

## 1. Introduction

Metasurfaces [1] are man-made structures consisting of repeating unit cells known as meta-atoms, which have gained notoriety relatively recently due to their property of exhibiting negative electric permittivity ϵ and magnetic permeability μ tensors at the same time [2,3,4]. Regardless of the sign of ϵ and μ, such designer-made structures exhibit a resonant response when subjected to external electromagnetic fields of an appropriate frequency [5]. The response can be tailored by an appropriate selection of the geometric shapes and sizes of the unit cell elements, as well as the nature of the materials chosen for them. For a given operating frequency, the resonant response can be determined from the change in reflection and transmission ratios of the wave intensity. For some metamaterial architectures, the change from low to high transmission is accompanied both by an inverse variation in reflection and a varying degree of absorbtion. Near-unity absorption is usually due to the instant discontinuities in the spatial phase of the unit cell, relative to the operating wavelength. Interfaces constructed from such structures have been extensively studied for gigahertz to terahertz detection [6], attenuation [7], and polarization control [8]. For frequencies drifting away from resonance, the metamaterials were responsible for the complete control of electromagnetic fields [9,10,11,12], together with a full series of new, exotic optical phenomena, such as regional cloaking [13], generalized reflection and refraction [14,15,16], polarization phase control [17,18], and a new class of exotic properties based on hyperbolic-tensor properties instead of conventional ellipsoid ones [19,20]. Real-time adjustment of the resonant response is made possible by the use of materials that are responsive to external fields, such as liquid crystal composites [21,22] layered on top of the unit cell, or the direct use piezoelectric unit cell components [23]. Due to the fact that they can both recreate all the properties of the conventional optical and terahertz materials and devices with equal or increased quality in resolution and integration capabilities, the range of applications of metamaterials includes all known applications in the optical and terahertz spectral regions, with various modifications resulting from the exotic set of properties of the meta-atoms. In the terahertz regime, the control of terahertz radiation in the G-band (100–300 GHz) has direct applications in terahertz sensing and imaging for security, defense, industrial quality control, spectroscopy, and more [24]. In addition, in spite of the creation of some differences in the spectral response, scalability of the architecture sizes can shift the working spectrum from the G-band up to the near infrared bands (30–60 THz) where similar architectures have been reported to serve as adequate sensors [25,26]. Further scaling of the metacell sizes allows operation in the optical regime, where similar crystalline structures have been reported to serve as polarization distributors and controllers [27]. An essential part of coherent control in these application is spectral filtering and transmission/reflection switching, which is accompanied by inherent absorption. This is due to the sharp discontinuities in the phase topology across the unit cell. When designing terahertz switches, the absorption coefficient has to be minimal, due to the fact that energy deposition in the switch leads to parasitic effects, such as loss of signal strength and uncontrollable frequency shifts in certain regions due to heat-induced material dilation. The resonant behavior of the cell can be attenuated significantly by designing new architectures that provide a smoother spatial phase distribution map across the cell. This can be achieved by using new three-dimensional etching and printing techniques of the cell elements, which have been previously used to design three-dimensional structures with resolutions well below the micrometer scale, and can ensure a practical realization of the envisioned architecture.

In this paper, we present theoretical investigations performed on a piezoelectric polymer-based architecture: a polyvinylidene fluoride (PVDF) cone-ring ensemble deposited on a silicon or a silica glass substrate. The reasons for choosing of this architecture and material combination are the following: Firstly, the PVDF polymer, the silicon and silica glass represent a readily-available and cost-effective solution, which have been previously investigated separately [28]. Secondly, the longitudinal piezoelectric effect of the PVDF provides active control on the spectral and phase responses by means of modifying the thickness of the cell elements [29]. The simulations that highlight the spectral response were performed in the terahertz G-band, in a window of interest ranging from 228 GHz to 232 GHz. The choice of the spectral window is justified by a multitude of applications in security, imaging and defense that have been successfully implemented in civil and military domains of interest. Specifically, there exists a wide range of airport scanners which are tuned for screening at a frequency *f* = 230 GHz, making our proposed architecture relevant for this type of application. The choice of such combination of materials for this window is justified by the favorable spectral properties of the individual components in the desired spectral range, together with the piezoelectric response of the PVDF, which is able to offer enhanced addressability. The results obtained highlight both the expansion of the architecture layout of the metamaterial from bi- to tridimensional, which is ensured by high-precision 3D printing techniques, such as two-photon polymerization [30], which boast an upper resolution limit of 150 nm in the current state-of-the-art [31]. The simplicity of the architecture, combined with the new degree of freedom created by the third dimension and the piezoelectric response of the PVDF make our proposed structure a viable candidate for an externally-addressable, high-quality switching device in the G-band, with a multitude of on-demand applications.

## 2. Structure Design and Simulation Parameters

**Figure 1:**
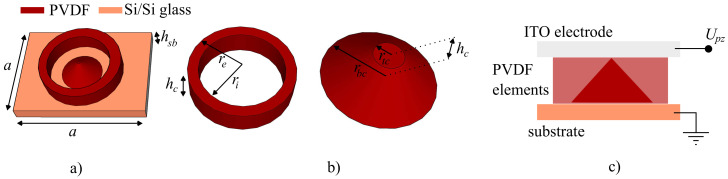
The proposed metasurface unit cell architecture, consisting of: (**a**) a PVDF ring-cone element placed on a silicon or silica glass substrate, depending on the configuration; (**b**) the geometric characteristics of the PVDF elements. (**c**) The envisioned direct piezoelectric effect setup, consisting of a transparent ITO layer by means of which an external sub-kilowatt value voltage is applied longitudinally across the unit cell, causing a variation in the PVDF elements height values $h_{c}$.

To simulate the response of the metamaterial unit cell, we assume that the incident wave has a plane wave solution, propagating in the *Oz* direction. The metacell is placed in the *Oxy* plane, in order to provide a response under normal incidence. In the design of the metacell structure, the priorities considered were spectral response and addressability. Due to the fact that the PVDF polymer exhibits a longitudinal piezoelectric effect, the structure exhibits symmetry along the propagation axis. This ensures that the external electric field will not modulate the terahertz field for frequencies above the MHz threshold and that for high-intensity terahertz fields, the polarization of the electric field in the *Oxy* will not induce localized asymmetries in the piezoelectric response of the medium. The proposed architecture is presented in Figure 1 and is formed of a concentric ring-cone section element system: the ring element has internal and external radii $r_{i}$ and $r_{e}$ respectively, while the cone section has the base radius $r_{bc}<r_{i}$, in order to provide a sufficiently large gap between the cone and ring, and the top radius $r_{tc}\leq r_{bc}$, in order to have the ability to transform the cone section from a cylinder to a full cone. The heights of the ring and cone $h_{r}$ and $h_{c}$ are equal and will serve as the externally-addressable variable. The ring-cone element system is sandwiched between a substrate and top plate of equal thickness $h_{sb}$. For both metacell samples we have designed so-called ’reference’ configurations, in which we established the fixed values for the geometry and placement on the substrate. From these reference configurations, we proceeded to analyze the tunable response of the metacell by varying one key parameter, while keeping the other fixed. We have performed separate studies for PVDF ring-cone elements sandwiched between either silicon or silica glass plates, which are treated with a relatively-thin (less than 200 nm) layer of indium-tin oxide (ITO). The presence of the ITO is mandatory in order to establish a longitudinal electric field, which is used to control the height of the system. The ITO layer is considered fully transparent to the optical wave and, therefore, does not interfere with the electromagnetic field. The configuration of the metacell is presented in Figure 1. The sizes of the elements, denoted generically as *r*, are first chosen as a rough estimate, according to the condition that requires that r<λ/5. Based on those estimations and the desired application, we have chosen the following values for the geometric parameters: *a* = 100 μm, *h_sb_* = 10 μm, *r_e_* = 30 μm, *r_i_* = 20 μm, *h_c_* = 10 μm, *r_bc_* = 15 μm, and *r_tc_* = 1.5 μm.

In terms of investigation conditions, the simulations were conducted using a commercial FDTD simulation software (Comsol Multiphysics − RF Module) in which both the electromagnetic response and the thermal energy deposition effects are taken into account. The incident plane wave has the electric component polarized along *Ox* and the magnetic component polarized across *Oy*. The sample under test has been situated within an air box with sizes a≥5λ, a condition that ensures that a steady-state solution of the optical field is recorded. The lateral sides of the air box were allocated a Floquet periodicity condition with a wave vector resulting from the calculated values of the wave vector within one cell. This ensures that the response characterizes a periodic structure along both axes *Ox* and *Oy*. The study was also set to trace out higher diffraction orders, which is in concordance with standard investigations. Regarding individual material properties, the bulk relative complex dielectric permittivity for the PVDF was $\epsilon_{PVDF}=9.55-j\cdot 0.05$, and conductivity $\sigma_{PVDF}=1.01$ mS/m. Moreover, to model the response to external electric fields, we have considered the inverse piezoelectric coefficient $d_{33}=49.6$ pm/V [32]. For the silica glass, the relative dielectric permittivity was taken as $\epsilon_{SG}=2.09$ and conductivity $\sigma_{SG}=10$ fS/m. The silicon parameters are $\epsilon_{Si}=11.7-j\cdot 0.01$ and conductivity $\sigma_{SG}=1.2$ pS/m. All of the components are considered non-magnetic (μ=1). With all these properties set, we use a fine-resolution discretization of the sample and the air box. Here, the mesh element sizes range from λ/10 in the regions where resolution is less important, such as lateral facets of the air box, to λ/50 in the regions where resolution becomes essential, such as the air/metamaterial interface and metacell elements. In addition, the solver was set to provide solutions of the field with a relative tolerance of $10^{-12}$, which was possible due to the appropriate choice of the mesh element size.

## 3. Simulations Sequence, Results, and Discussions

The simulations were conducted to highlight the spectral properties of both unit cell configurations (silica glass vs. silicon substrates), under variation of selected geometric sizes in the following manner: The discrete parameter values were selected to fit two criteria: first criterion is that the unit cell configuration has to be experimentally constructed with reasonable accuracy, especially in the third dimension. The other criterion is that the spectra should exhibit significant modification from one parameter value to another. Lastly, the variations in geometric size induced by the longitudinal piezoelectric effect have to be consistently chosen as to recreate experiment. With all these considerations in mind, we have first conducted simulations in which the conical structure was modified from a completely cylindrical shape to a conical one by modifying the values of the top radius $r_{tc}$. This study was performed to demonstrate that due to the linear spatial phase gradient of the conical structure, the transition from a reflective to a transmitting metastructure is performed with the least amount of loss. After this, we set the conical shape as the reference, and performed a study of the spectral response as a function of both ring and cone height $h_{c}$ for multiple construction configuration values. Based on the results and the desired application, we have chosen an appropriate value for $h_{c}$ to serve as the reference unit cell configuration. Lastly, to highlight the changes induced by the piezoelectric effect, we have modified the values of $h_{c}$ to have values around the reference unit cell value. The values of $h_{c}$ are dictated both by the piezoelectric coefficient of PVDF and experimentally feasible voltages across the unit cell, which are typically below 1 kilovolt. During one study, all parameters which are not considered of interest were kept at constant values. Moreover, due to the fact that the investigations conducted at this step do not intend to provide an optimization path of a certain application of the proposed architecture (e.g., optical field switching, polarization control, selective absorption), the unit cell parameters remain unchanged from one study to another, in order to maintain a coherent and relevant reference point.

### 3.1. Cylinder-to-Cone Structure Simulations

**Figure 2:**
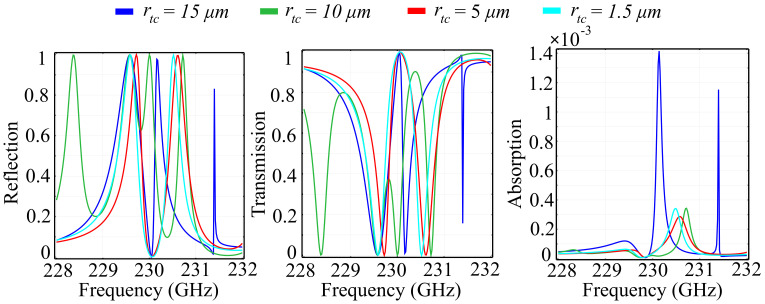
Simulated reflection, transmission, and absorption spectra for the silica glass reference cell in which the top radius of the inner-ring cylinder $r_{tc}$ is varied between fixed values, in a transition from a cylinder to a cone structure.

**Figure 3:**
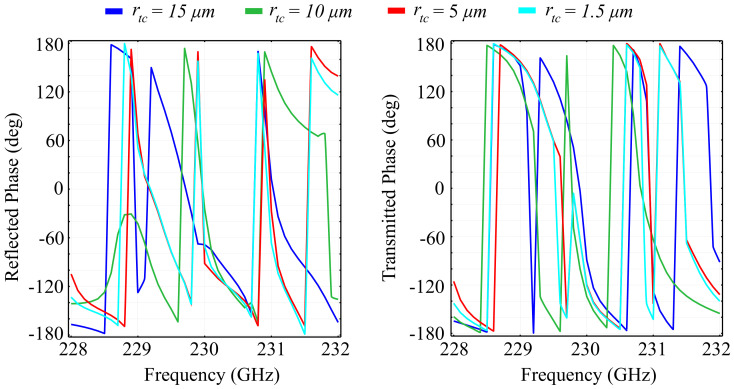
Simulated phase for the reflected and transmitted waves in the silica glass-based configuration, in which the top radius of the inner-ring cylinder $r_{tc}$ is varied between fixed values, in a transition from a cylinder to a cone.

To highlight the response arising from both continuous and discontinuous spatial phase profiles induced by the ring-cone structure, as well as to validate our necessity for a spatial phase gradient in the *Oz* direction, we have performed simulations on a reference cell in which the top radius of the cone was varied between fixed values. The values were chosen in such a way as to indicate the step-by-step transition from a cylindrical to a conical shape of the inner-ring component. The reflected, transmitted, and absorbed wave components are presented in Figure 2. In addition, Figure 3 presents the associated phase of the reflected and transmitted waves, as well as offers valuable information on the polarization control of the desired wave.

The obtained results can be interpreted as follows: The reference silica glass configuration exhibits a strong response in the spectral region of interest with multiple transitions from a reflecting to a transmitting metastructure, as a function of frequency. The reflection behavior is, as expected, alternating with the transmission behavior, and the range of both coefficients is complete, between zero and unit values. The observed reflection peaks for $r_{tc}=15$ μm, which corresponds to a cylindrical shape of the center element occur at frequencies *f* = 229.6 GHz, 230.5 GHz, and 231.5 GHz. An interesting result is the presence of the extra reflection peak at 231.4 GHz, which is not encountered for any of the conical shapes of the center element. The transmission picture mirrors the reflection for all configurations, and for the cylinder shape of the center element the transmission peaks occur at *f* = 230.05 GHz and 231.35 GHz, with the latter peak occurring just before a sharp dip in the transmission curve. The absorption behavior varies significantly as the cell configuration switches from a cylinder to a cone shape of the center element. For the $r_{tc}=15$ μm configuration, corresponding to a cylindrical shape of the center element, the absorption exhibits two peaks at 230.1 GHz and 231.37 GHz, which correspond to the inflection points of the transmission and reflection curves, as they perform their respective variations from minimum to maximum, and vice-versa. The values of the absorption coefficient are low, with less than 0.2% of the incident wave being absorbed in the structure. As $r_{tc}$ decreases and the center element shape becomes increasingly conical, the reflection and transmission spectra suffer significant modifications: For the $r_{tc}=10$ μm configuration, two new reflection maxima are observed at 228.4 GHz, corresponding with a transmission minimum at the same frequency, and associated with a negligible absorption. The other configurations depicted in Figure 2 maintain roughly the same tendency as the cylindrical centerpiece configuration, but with shifted maxima and minima both in reflection and transmission. In addition, for all conical-shape centerpiece element, the absorption coefficient maintains values four times smaller than the cylinder centerpiece configuration, which makes the conical configuration suitable for switching applications. We can also see that the $r_{tc}=5$ μm, which corresponds to a non-ideal conical shape of the center element offers the smoothest transition from a reflecting to a transmitting behavior, with the smallest value of the absorption peak. This condition relaxes the fabrication condition of the conical shape for the center element. In addition, a possible explanation for the fact that, surprisingly, the $r_{tc}=1.5$ μm exhibits higher absorption than the $r_{tc}=5$ μm configuration is that the point-like surface at the tip of the cone acts as a nano-resonator, increasing absorption. Regarding the phase of the reflected and transmitted beam, it is notable that all configurations exhibit an almost complete, 2π phase range, making the proposed architecture configurations suitable for polarization generation and control across the entire Poincaré sphere. The cylinder-shaped center element configuration offers six phase shifts with varying angular range. The most accentuated shift, spanning the full 2π range occurs at *f* = 228.4 GHz, which corresponds to the inflection point of the reflection and transmission curves. The other phase shifts span between $240^{{}^{\circ}}$ and $320^{{}^{\circ}}$, which, even though less-than-ideal, still offer superior quality phase control. The phase-shift tendency is maintained for the other configurations, with a certain frequency displacement. This leads to the possibility of control of the phase shift as a function of the top radius of the center element, adding a degree of versatility to the proposed architecture.

**Figure 4:**
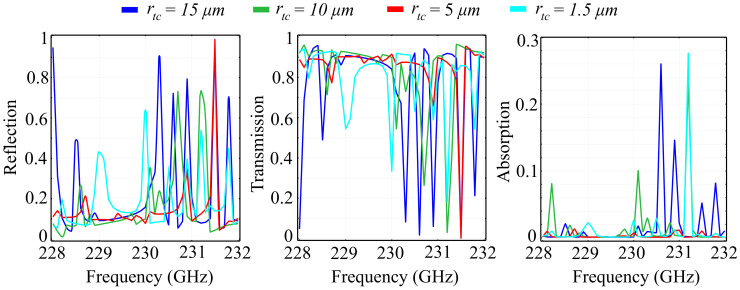
Simulated reflection, transmission, and absorption spectra for the silicon substrate configuration, in which the top radius of the inner cylinder ring $r_{tc}$ is varied between fixed values, in a transition from a cylinder to a cone structure.

**Figure 5:**
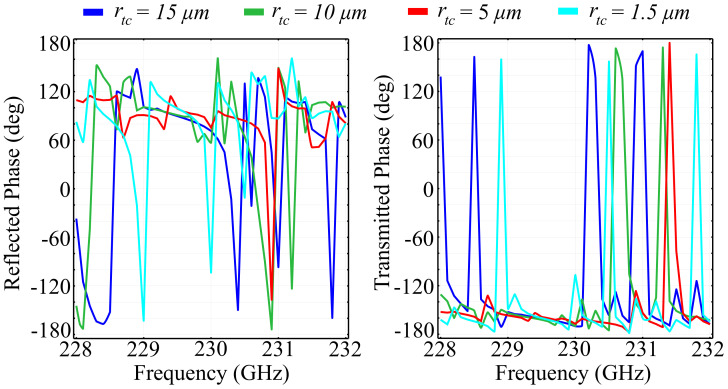
Simulated phase of the reflected and transmitted waves in the silicon substrate configuration, in which the top radius of the inner-ring cylinder $r_{tc}$ is varied between fixed values, in a transition from a cylinder to a cone structure.

For comparison, we have also conducted simulation of the spectral response of the silicon-based metacell. The results for the obtained reflection, transmission, and absorption coefficients are presented in Figure 4. In addition, to highlight the polarization control properties, we have simulated the phase of the reflected and transmitted waves in the silicon-based unit cell configuration. The result is presented in Figure 5.

The results obtained highlight the following properties: Firstly, the silicon substrate configuration exhibits a more abrupt transition in the spectral response than the silica glass. This behavior is observed both in reflection and transmission, with multiple reflection peaks occurring across the desired spectrum. For the $r_{tc}=15$ μm configuration, that corresponds to a cylindrical shape of the center element, the reflection peaks are obtained at 228 GHz, 228.5 GHz, 230.3 GHz, 230.55 GHz, 230.9 GHz, 231.5 GHz, and 231.8 GHz. The peaks, however, have a maximum reflection value of 0.9 instead of 1. For the conical-shape center element configuration, the reflection peaks are shifted in frequency, and there exists a maximum peak value of almost unity for the reflection coefficient, obtained for the $r_{tc}=5$ μm configuration. The transmission behavior of the configurations mirror the reflection curves, with dips occurring at roughly the same frequencies as the reflection peaks. This behavior is expected for surfaces that do not exhibit significant absorption, which is the case here. However, the absorption coefficient for the silicon substrate configuration is at least a hundred times higher than its silica glass counterpart, with absorption peaks reaching 0.25 of the total wave for specific frequencies. Again, the least amount of absorption is exhibited by the $r_{tc}=5$ μm configuration, which is counterintuitive, but can again be explained by the fact that smaller top radii of the center element act as resonance plasmons and, therefore, contribute to higher absorption in the material. The absorption curves justify this explanation, as the highest absorption coefficient is remarkably obtained by the $r_{tc}=5$ μm configuration. Regarding the phase accumulated by the reflected and transmitted waves, the results show that the silicon-based configuration also has the possibility to control the polarization of the transmitted wave across the full 2π angular range. However, the reflected wave has a less-than-ideal phase control, with a $320^{{}^{\circ}}$ angular range. The phase shifts are increasingly abrupt as the frequency increases, for all tested $r_{tc}$ configurations, and appear at the inflection points of the transition from the low- to high-reflection, and vice-versa.

### 3.2. Ring-Cone Height Simulations

**Figure 6:**
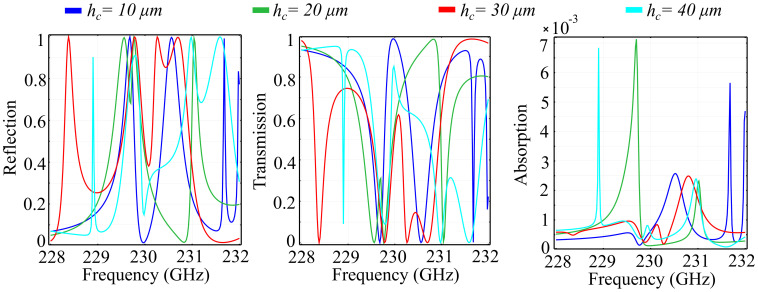
Simulated reflection, transmission, and absorption spectra for fixed ring-cone height ($h_{c}$) values, in the silica glass substrate unit cell configuration.

**Figure 7:**
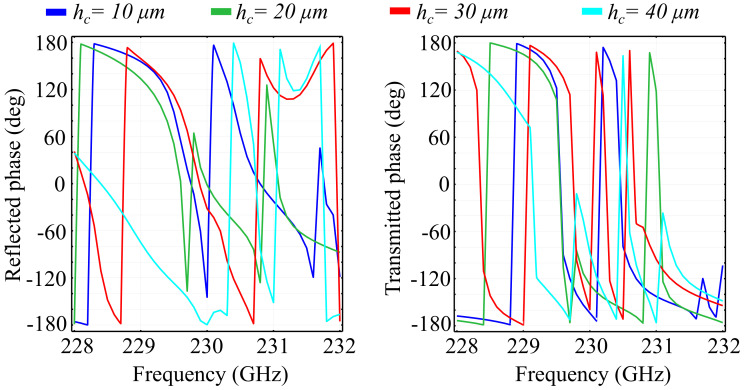
Associated phase for the reflected and transmitted waves, for fixed ring-cone height ($h_{c}$) values in the silica glass substrate configuration.

More so than in the case of two-dimensional unit cell configurations, the introduction of the *Oz* axis as a degree of freedom mandates that the investigations conducted are also focused on the changes imparted by the height of the unit cell elements $h_{c}$. Here, the chosen element height is non-negligible with respect to the wavelength, with considerable predicted impact on the spectral response. The study was conducted for several fixed value configurations of the element height, with the added mention that, for all configurations, we maintained an equal height for both the ring and the cone structures. This condition was imposed by practical limitations, due to the fact that etching of different element heights, while still feasible, imposes considerable technological requirements in terms of resolution and materials. As before, we have conducted our investigations on the two substrate types, namely silica glass and silicon. The simulation results obtained for the PVDF/silica glass in the standard configuration are presented in Figure 6, and the associated phase for the above cell configurations is presented in Figure 7.

The results obtained indicate the following properties: Firstly, as predicted, the spectral response changes dramatically with the element height $h_{c}$. For some frequencies, an increment of 10 μm in the height corresponds to a complete change of behavior, from a transmitting to a reflecting unit cell. The reflection spectra highlights the existence of multiple reflection peaks in the desired window, for all configurations. In addition, for all configurations, the transition from zero to full reflection (and inversely from unity to zero in transmission) is relatively lean, suggesting there are no resonances that can be attributed to locality. This conclusion is supported by the obtained absorption values, which are less than 1% of the incident wave. In the $h_{c}=20$ μm, we observe a modified spectral response, with less numerous reflection peaks and a relatively-wide (1 GHz ) transition slope, from 230 GHz to approximately 231 GHz. The $h_{c}=30$ μm configuration exhibits a more pronounced reflective behavior, with a new peak obtained at *f* = 228.6 GHz. Unlike the lower-valued $h_{c}$ configurations, the dip in reflection at around 230 GHz does not attain near-zero values. These values, however, are attained in a band between 231.6 GHz and 231.8 GHz, where all the other configurations reflect more than 10% of the incoming wave. The $h_{c}=40$ μm configuration exhibits a sharp near-unity reflection peak at 228.9 GHz, together with a quasi-linear transition slope between 230 GHz and 231 GHz. Full reflection in this configuration is obtained for 231 GHz and 231.5 GHz. In addition, the transmission behavior mirrors the reflection behavior almost perfectly, a behavior supported by the low absorption values.

Regarding the phase of the reflected and transmitted waves, the obtained results indicate that all PVDF/silica glass configurations exhibit full 2π control of the wave, with sharp frequency-dependent transitions. As $h_{c}$ increases, the phases of the reflected and transmitted waves maintain their general tendency as in the reference configuration, exhibiting sharp transitions from a negative to a positive value at a certain frequency, which are then followed by a slower, smoother transition back into the negative domain, at a different value than the previous. Apart from this behavior, the $h_{c}=30$ μm and $h_{c}=40$ μm configurations exhibit a relatively-reduced phase variation in the 230.8 GHz–231.9 GHz and 231 GHz–231.4 GHz bands, respectively. The phase of the transmitted wave exhibits a similar behavior as the translation phase, with sharper transitions from $+108^{{}^{\circ}}$ to a negative value across the spectral window. When increasing the values of $h_{c}$, we can see that the transitions in phase have their frequencies shifted to the left and to the right, with respect tot the reference configuration, depending on the frequency considered and the $h_{c}$ value. A notable result is that, in the case of the $h_{c}=30$ μm configuration, an extra shift is observed at 230.6 GHz, while, for the $h_{c}=40$ μm, the shift at 230.5 GHz is accompanied by two minor shifts at 229.8 GHz and 231 GHz.

**Figure 8:**
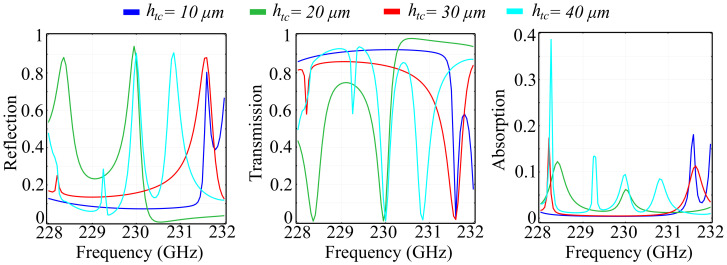
Simulated reflection, transmission, and absorption coefficients for fixed ring-cone height ($h_{c}$) values in the silicon substrate unit cell configuration.

**Figure 9:**
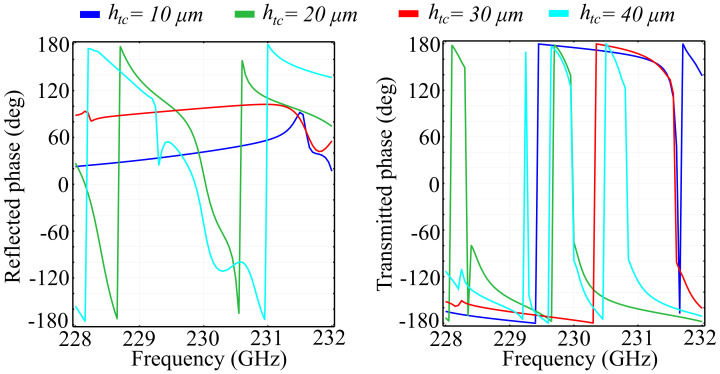
Simulated phase of the reflected and transmitted waves for fixed ring-cone height ($h_{c}$) values in the silicon substrate unit cell configuration.

Just as before, we have performed a similar investigation for the silicon substrate-based unit cell, under the same simulation conditions and parameter values. The study was performed to highlight the spectral properties of both architectures in a comparative manner. The reflection, transmission, and absorption coefficients are presented in Figure 8, and the associated phases of the reflected and transmitted waves are presented in Figure 9:

The simulated results show that, in this configuration, the behavior differs considerably from the silica glass substrate configuration. Firstly, the reflection coefficient for the reference cell configuration has a low value range (less than 0.2) across most of the spectral range, and exhibits a sharp rise at 231.5 GHz, to a value of 0.8 of the incident wave. For increasing fixed values of $h_{c}$, the spectral curve changes dramatically, with new reflection peaks appearing within the frequency window. Most notably, in the $h_{c}=20$ μm configuration, the reflection coefficient attains a minimum value of approximately 0.02 at a frequency *f* = 230.4 GHz which is mirrored by a transmission value of 0.97 at the same frequency. This configuration also exhibits two reflection peaks, the first at 228.4 GHz with a value of 0.88, and the second at 229.9 GHz, with a value of 0.94. The transmission curves exhibit several dips to zero transmission across the spectral window, but it is noteworthy that, in the reference configuration, the metasurface is transmitting across the vast majority of the spectral interval, with values between 0.8 and 0.92. Regarding absorption, unlike the silica glass substrate configuration, here, we have obtained significant absorption, with a value of 0.38 in the $h_{c}=40$ μm, obtained at 228.3 GHz. The rest of the absorption peaks for all other configurations have their values below 0.2, which still represents a factor of 50 above the absorption values obtained in the silica glass substrate configuration.

The results obtained for the accumulated phase of the reflected and transmitted waves in the silicon substrate configuration also offer interesting insight: In the reference configuration, the metasurface has limited polarization control capabilities, with a frequency-phase response of $90^{{}^{\circ}}$. The same type of behavior is repeated for the $h_{c}=30$ μm variant, which also exhibits a relatively-flat response. The $h_{c}=20$ μm and $h_{c}=40$ μm configurations, however, exhibit a full 2π phase response across the desired spectrum: the sharp transitions from $-180^{{}^{\circ}}$ to $+180^{{}^{\circ}}$ are followed by relatively-smooth transitions. For the $h_{c}=20$ μm configuration, the sharp transitions occur at 228.75 GHz and 230.6 GHz, while, for the $h_{c}=40$ μm, these transitions can be observed at 228.3 GHz and 231 GHz. Due to the fact that, unlike some other architectures where the response can be inferred by frequency shifting, the reflection response of the metasurface is not following a given tendency, the specific conditions of the applications are the ones that decide the sizes of the ring-cone structure height. In transmission, the response curves are following the same general tendency regardless of the configuration used, and they are exhibiting full 2π polarization control, with sharp transitions from $-180^{{}^{\circ}}$ to $+180^{{}^{\circ}}$ and smoother transitions in the opposite direction, as the frequency increases. The number of transition peaks, as well as the bandwidth of the smooth transitions are dictated by the sizes of $h_{c}$, just as in the case of reflection.

### 3.3. Piezoelectric Effect Simulations

**Figure 10:**
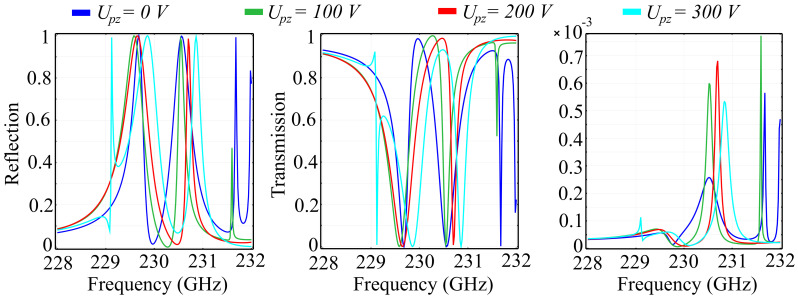
The reflected, transmitted, and absorbed waves for fixed values of the piezoelectric voltage applied longitudinally across the unit cell in the silica glass substrate unit cell configuration.

**Figure 11:**
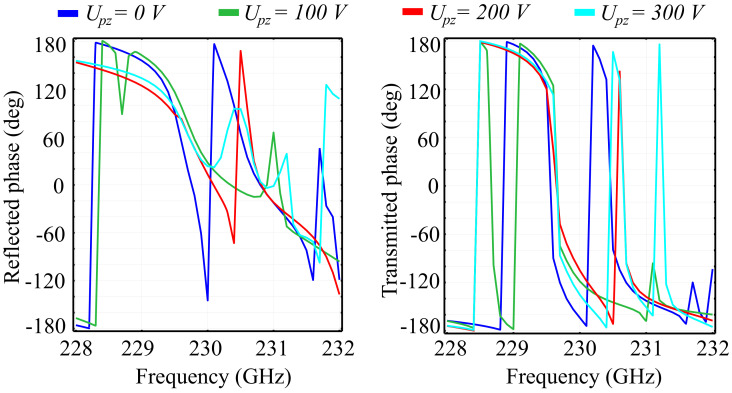
Simulated phase of the reflected and transmitted waves associated to the piezoelectric voltages applied longitudinally across the unit cell in the silica glass substrate unit cell configuration.

We have also performed simulations that highlight the response of the metasurface cell to the piezo-electrically induced variations in the height of the ring and cylinder for both silica glass and silicon substrate configurations. Based on the value of the longitudinal piezoelectric constant, the simulated height variations correspond to sub-kilowatt level voltages, ranging from $U_{pz}$ = 0 V to $U_{pz}$ = 300 V. Such voltages can be applied to the PVDF element in the proposed architectural dimensions without any electrical discharges between the dielectric elements. The probability for discharge, however, increases as the gaps between the conducting $TiO_{2}$ layers decrease. The longitudinal piezoelectric effect of the PVDF elements is included in the simulation by relating the ring-cone structure height $h_{c}$ to the applied voltage through the longitudinal piezoelectric coefficient $d_{33}$. Due to the fact that the anisotropic piezoelectric coefficients have their values more than a hundred times smaller than $d_{33}$, the induced transverse piezoelectric effect can be neglected here. The results for the reflected transmitted and absorbed wave ratios in the silica glass substrate configuration are presented in Figure 10. In addition, the associated phase of the reflected and transmitted waves are presented in Figure 11.

Based on the results obtained, the following considerations can be made: Firstly, the unit cell exhibits a relatively-high sensitivity to voltage-induced variations of $h_{c}$, with a significant modification of the spectral response between the fixed-values of the voltage. Due to the fact that the transformation of the spectral response between two voltage values is characterized by a continuous function, a morphing of the spectral response between the two voltage values can be discussed. This behavior offers promising spectral tuning possibilities obtainable through a continuous increment of the applied voltage values. Secondly, as expected, when increasing the applied voltage, the reflection peaks shift from their reference cell configuration values. The reference configuration exhibits two reflection peaks at 231.6 GHz and 231.9 GHz, which have no correspondents in the $U_{pz}=200$ V and $U_{pz}=300$ V configurations. In the $U_{pz}=100$ V configuration, the reflection peak observed at 231.6 GHz in the reference cell configuration is shifted to 231.5 GHz and significantly attenuated, while the reflection peak at 231.9 GHz in the reference cell configuration has no correspondent. In the $U_{pz}=300$ V configuration, an additional reflection peak is observed at 229.1 GHz, which also has no correspondent in the other cell configurations. The transmission behavior mirrors the reflection behavior almost perfectly, which can also be quantitatively observed in terms of absorption. Here, the absorption levels are fairly insignificant for the low-power regime, making the silica glass-based unit cell a viable candidate for tunable switching applications.

Regarding the accumulated phases of the reflected and transmitted waves, it can be observed that the application of the external voltage negatively affects the phase response across the spectrum. For the reflected wave, the reference cell exhibits a full 2π phase response across the spectrum window, primarily expressed by the shift observed at 228.3 GHz. This behavior is mimicked almost entirely by the $U_{pz}=100$ V cell configuration, with some attenuation and peak shifts. The $U_{pz}=200$ V and $U_{pz}=300$ V configurations, however, lose the 2π shift, and exhibit a relatively-monotonous transition from $+180^{{}^{\circ}}$ to values between $-100^{{}^{\circ}}$ and $-120^{{}^{\circ}}$, depending on the configuration. In transmission, the application of the external field does not influence the 2π phase transitions, only shifts them with respect to the reference cell configuration. A noteworthy observation is the appearance of an additional peak in the $U_{pz}=300$ V configuration at 231.3 GHz, with no correspondent in the other configurations.

**Figure 12:**
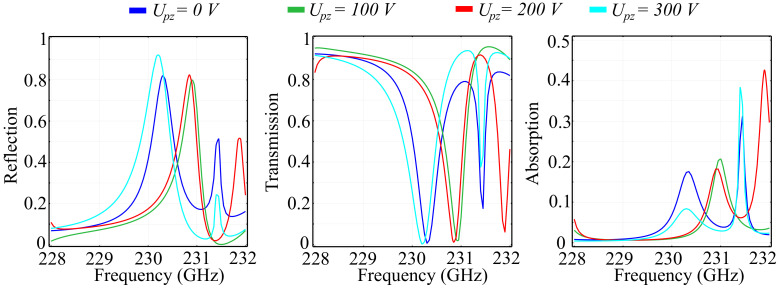
The reflected, transmitted, and absorbed waves for fixed values of the piezoelectric voltage applied longitudinally across the unit cell, in the silicon substrate unit cell configuration.

**Figure 13:**
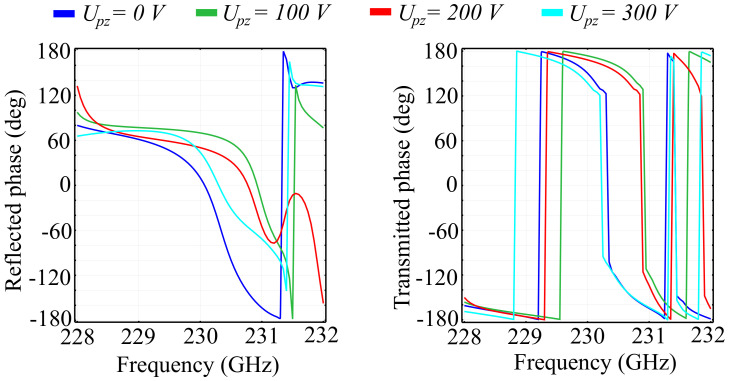
Simulated phase of the reflected and transmitted waves associated to the piezoelectric voltages applied longitudinally across the unit cell in the silicon substrate unit cell configuration.

Just as before, we performed a comparative study for the silicon substrate configuration for the same values of the applied voltage $U_{pz}$. The results obtained for the reflection, transmission, and absorption coefficients are shown in Figure 12, and the results for the accumulated phase of the reflected and refracted beams are shown in Figure 13.

The simulated results allow for the following considerations to be made: Firstly, by comparison to the silica glass substrate configuration, the reflection and transmission responses have much leaner transitions between a reflecting and transmitting behavior. Secondly, the curves follow the same tendency but with relatively-high sensitivity across the voltage values. This can be reflected both in the shift in frequency obtained for the various $U_{pz}$ values and in the significant modification of the maximal and minimal values in reflection, transmission, and absorption. This sensitivity makes the silicon substrate configuration suitable for high precision tuning with lower-valued voltage values. For both reflection and transmission, the maximum values are considerably less than unity, with maxima reaching a value of 0.9 at 230.3 GHz in the $U_{pz}=300$ V configuration for reflection, and a value of 0.94 at 231.3 GHz in the $U_{pz}=100$ V configuration. In addition, this configuration exhibits a significant resonant response, with absorption peaks reaching values of up to 0.43 at 231.6 GHz for the $U_{pz}=200$ V configuration. The resonant response of the silicon substrate configuration makes it a viable candidate for the realization of frequency-selective surfaces in this spectral window.

Regarding the phase response of the reflected and transmitted waves, the silicon substrate configuration exhibits a 2π phase control over the spectral window of interest. In reflection, we can observe a moderately-smooth transition from around $120^{{}^{\circ}}$ at 228 GHz to $-180^{{}^{\circ}}$ at frequencies around 231.2 GHz, depending on the applied $U_{pz}$ value. In addition, the swift transition from $-180^{{}^{\circ}}$ to $180^{{}^{\circ}}$ encountered at those frequencies is no longer observable for the $U_{pz}=200$ V. This behavior can lead to another degree of control over the polarization of the reflected wave. The phase response of the transmitted wave follows the same tendency, with abrupt transitions from $-180^{{}^{\circ}}$ to $180^{{}^{\circ}}$ and back, while also having a relatively-small smooth transition band. The applied voltage does not induce significant modifications in the shape of the response, rather it only shifts the frequencies at which the transitions take place as a function of the applied voltage. The shift is not monotonous, which implies that there exist pairs of values for $U_{pz}$ that once applied to the structure will produce almost identical results in transmission over the spectral window. This type of behavior can be used for building optical differential voltage sensors, that sense the shift in the response at a given point based on the difference between the pair of voltages that produce identical responses.

### 3.4. Nonlocal Field Characterization

**Figure 14:**
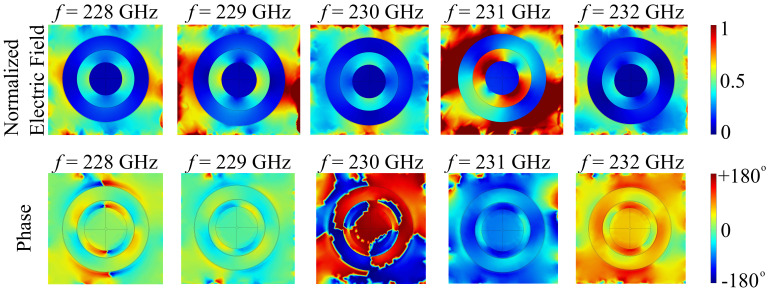
Simulated electric field and in-plane nonlocal phase distributions across the cell element for the silica glass substrate configuration.

To assess the nonlocal properties of the cell surface, we have also performed simulations of the electric field and spatial phase distributions at the interface between the cone and ring elements and the substrate. These coordinates have been chosen due to the fact that here, the influence of the artificial spatial phase distribution imparted by the unit cell elements is the highest. The simulations are performed at frequency values chosen in order to perform a scan of the spectral region of interest. The results are presented in Figure 14.

The results show a significant nonlocality of the metasurface, with the electric field being scattered preferentially across the metasurface unit cell. For 228 GHz, the field distribution is scattered rather uniformly over the cell, but it is noted that it has zero value across the polymer elements, even though they are dielectric, and can support nonzero field modes. The associated nonlocal phase distribution is symmetric, and ranges from $-180^{{}^{\circ}}$ to $+180^{{}^{\circ}}$, with a smooth transition across most of the geometry. At 229 GHz, the normalized field distribution remains symmetric, and it gains considerable amplification at the lateral sides of the unit cell, while keeping an almost zero value across the ring and cone polymer elements. The nonlocal phase domain is reduced at this frequency, ranging from $-90^{{}^{\circ}}$ to $+90^{{}^{\circ}}$ for the greater part of the surface. At 230 GHz, the field distribution changes symmetry from a lateral to an azimuthal direction, with near-zero field values across the ring and cone polymer elements. The nonlocal phase, however, exhibits either maximal or minimal values, with strong, asymmetrically displaced transitions across the surface. At 231 GHz, the symmetry of the field distribution is diagonal, and there is significant field distribution across the ring and cone cell elements. The phase, however rescinds to values near $-180^{{}^{\circ}}$ for the most part of the cell. At 232 GHz, the field distribution symmetry is anti-diagonal, with a return of near-zero values across the polymer cell elements. The phase is strongly biased towards $+180^{{}^{\circ}}$ for the most part of the surface, and exhibits a smooth transition to $0^{{}^{\circ}}$ in the lateral areas of the cell.

**Figure 15:**
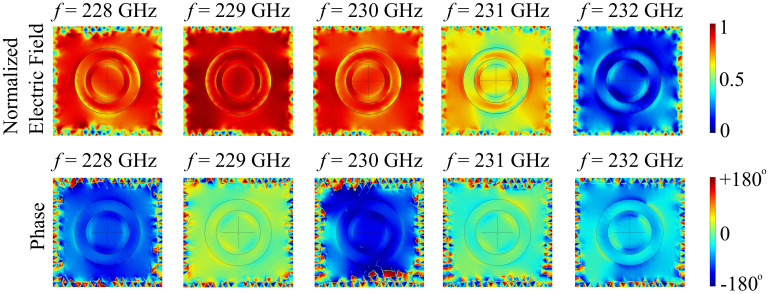
Simulated electric field and in-plane nonlocal phase distributions across the cell element for the silicon substrate configuration.

Just as before, we conducted a comparative study on the nonlocal properties of the silicon substrate configuration. The results are shown in Figure 15.

It is clear from the results that the uneven distribution of the electric field and spatial phase on the surface of the unit cell promotes a significant nonlocal behavior of the silicon substrate configuration of the metasurface. At 228 GHz, the electric field is relatively uniformly distributed across the surface with the exception of the PVDF ring and cylinder edges. The associated phase follows the same uniform distribution in the central part of the cell, with abrupt transitions in the ring-cone gap. At 229 GHz, the field distribution suffers an increase in both area of uniform distribution and value, while the associated phase also shifts from mostly negative values to near-zero values. At 230 GHz, the field distribution maintains roughly the same distribution at slightly lower field values, but the phase is shifted back to near $-180^{{}^{\circ}}$ values in the central part of the cell. At 231 GHz, the field distribution becomes increasingly nonlocal, with some parts of the surface having higher field values than others. The field distribution also attains a certain symmetry along the lateral axis. The associated phase maintains relative uniformity across the surface, with near-zero values and abrupt shifts near the edges of the ring and cone elements. Finally, at 232 GHz, the field distribution is radically modified with respect to the other configurations, the majority of the field being concentrated near the edges of the cell, therefore displaying significant nonlocality. The associated phase is also distributed in a nonuniform manner, with shifts at the edges of the polymer elements.

## 4. Conclusions

In this paper, we have investigated the spectral response of a piezoelectrically-addressable polyvinylidene fluoride (PVDF) ring-cone metasurface in the terahertz G-band, in a window that is commonly used for security applications and terahertz imaging. The unit cell was deposited on two commonly used substrates—silica glass and silicon—, and their spectral responses were investigated both individually and comparatively. The results show a complete control of the electric field properties and on-demand tunability via the piezoelectric effect that is exhibited by the PVDF elements. In addition, depending on the substrate and geometries used, the metasurface can be used for high quality switching between reflection and transmission, phase control and significant resonant behavior within the spectral window of interest. The spectral response is significantly modified based on the substrate and small variations in the sizes of the architecture elements, which highlights an enhanced versatility of the metasurface without any costly modification to the structure. This implies that the metasurface can be used in various terahertz applications, such as imaging, switching, detection, and control of the terahertz electromagnetic fields in the G-band window of interest, without any significant changes to the manufacturing process. The piezoelectric effect exhibited by PVDF offers active control capabilities, making it possible for the same metasurface configuration to be used in multiple applications by only varying the externally-applied voltage.

## Data Availability

Data sharing not applicable.
